# *Oscillatoria* sp. as a Potent Anti-phytopathogenic Agent and Plant Immune Stimulator Against Root-Knot Nematode of Soybean cv. Giza 111

**DOI:** 10.3389/fpls.2022.870518

**Published:** 2022-05-26

**Authors:** Rehab Y. Ghareeb, Nader R. Abdelsalam, Dahlia M. El Maghraby, Mahmoud H. Ghozlan, Eman EL-Argawy, Reda A. I. Abou-Shanab

**Affiliations:** ^1^Department of Plant Protection and Biomolecular Diagnosis, Arid Lands Cultivation Research Institute (ALCRI), City of Scientific Research and Technological Applications (SRTA-City), Alexandria, Egypt; ^2^Department of Agricultural Botany, Faculty of Agriculture, Saba Basha, Alexandria University, Alexandria, Egypt; ^3^Department of Botany and Microbiology, Faculty of Science, Alexandria University, Alexandria, Egypt; ^4^Department of Plant Pathology, Faculty of Agriculture, Damanhour University, Damanhour, Egypt; ^5^Department of Environmental Biotechnology, Genetic Engineering and Biotechnology Research Institute (GEBRI), City of Scientific Research and Technological Applications (SRTA-City), Alexandria, Egypt; ^6^Biotechnology Institute, College of Biological Sciences, University of Minnesota, St. Paul, MN, United States

**Keywords:** blue-green algae, *Meloidogyne incognita*, water extract, methanol extract, differential display-PCR, gene expression, soybean

## Abstract

**Background:**

Plant-parasitic nematodes are one of the major constraints to soybean production around the world. Plant-parasitic nematodes cause an estimated $78 billion in annual crop losses worldwide, with a 10–15% crop yield loss on average. Consequently, finding and applying sustainable methods to control diseases associated with soybean is currently in serious need.

**Methods:**

In this study, we isolated, purified, characterized, and identified a novel cyanobacterial strain *Oscillatoria* sp. (blue-green alga). Based on its microscopic examination and 16S rRNA gene sequence, the aqueous and methanolic extracts of *Oscillatoria* were used to test their nematicidal activity against *Meloidogyne incognita* hatchability of eggs after 72 h of exposure time and juvenile mortality percentage *in vitro* after 24, 48, and 72 h of exposure time and reduction percentage of galls, eggmass, female number/root, and juveniles/250 soil. Also, the efficacy of the extract on improving the plant growth parameter and chlorophyll content under greenhouse conditions on soybean plant cv. Giza 111 was tested. Finally, the expression of PR-1, PR-2, PR-5, and PR15 (encoding enzymes) genes contributing to plant defense in the case of *M. incognita* invasion was studied and treated with *Oscillatoria* extract.

**Results:**

The aqueous and methanolic extracts of *Oscillatoria* sp. had nematicidal activity against *M. incognita*. The percentage of mortality and egg hatching of *M. incognita* were significantly increased with the increase of time exposure to *Oscillatoria* extract 96.7, 97, and 98 larvae mortality % with S concentration after 24, 48, and 72 h of exposure time. The aqueous extract significantly increased the percentage of Root-Knot nematodes (RKN) of egg hatching, compared with Oxamyl and methanol extract at 96.7 and 97% after 72 h and 1 week, respectively. With the same concentration in the laboratory experiment. Furthermore, water extracts significantly reduced the number of galls in soybean root, egg masses, and female/root by 84.1, 87.5, and 92.2%, respectively, as well as the percentage of J2s/250 g soil by 93.7%. Root, shoot lengths, dry weight, number of pods/plant, and chlorophyll content of soybean treated with *Oscillatoria* water extract were significantly higher than the control increasing by 70.3, 94.1, 95.5, and 2.02%, respectively. The plant defense system's gene expression was tracked using four important pathogenesis-related genes, PR-1, PR-2, PR-5, and PR15, which encode enzymes involved in plant defense.

**Conclusions:**

*Oscillatoria* extract is a potential nematicide against root-knot nematode invasion in soybean.

## Introduction

Soybean (*Glycine max* L.) has been regarded as one of the most important summer legume crops for human and animal diets worldwide (Adami and Leskovšek, 2021; Abebe, 2022), having served as an important source of dietary protein and oil for the past 20 years (Akmalovna, 2022). In addition, its consumption has outpaced its production. This trend is expected to continue with increased demand for soybeans reaching 300 million tons in 2020 (Lima et al., 2017). Soy is used for food and feed, as a raw material in the vegetable oil industry and for other purposes such as the manufacture of plastics, lubricants, candles, varnishes, soaps, biodiesel, and lecithin (Fontana and Partridge, 2015; Xu et al., 2022), the yields of which accounted for nearly 60% of the world's oilseed production in 2018 (Yao et al., 2020).

Soybean seeds contain ~19–22% oil and 30–50% protein (Yao et al., 2020; Sun et al., 2022), and they are parasitized by a variety of pests and diseases, the most serious of which are plant-parasitic nematodes (PPNs) that represent a major threat (Campbell and Glatz, 2009; Lima et al., 2017). The root-knot nematode Meloidogyne Göldi, 1892 (Rhabditida: Meloidogynidae) is one of the most economically important plant pathogens and causes severe damage to most of the crops worldwide (Archidona-Yuste et al., 2018; Gareeb et al., 2019; Detrey et al., 2022). To date, ~4,100 species of plant-parasitic nematodes have been identified, with a small number of genera considered to be causing major plant diseases and others specialized to a smaller number of crops, both of which have a significant impact on economically important crops. Plant nematodes are expected to cause a global yield loss of 12.3% ($157 billion) (Singh et al., 2015; Poveda et al., 2020), which is greater than the harm caused by invading insects (~US$70 billion (Badenes-Pérez and López-Pérez, 2018; Poveda et al., 2020). The root-knot nematodes Meloidogyne spp. are of great economic and social importance, being serious plant pathogens worldwide distribution infecting a wide range of crops causing losses up to 80% in heavily infested fields with average yield losses ranging from 28 to 68% (Chaudhary et al., 2011).

Aside from PPN, a variety of insect pests attack soybean, with stem-feeding pests posing the greatest threat. Soybean is a plant that is known to be infected with root-knot nematodes, especially *Meloidogyne* species (Chaudhary et al., 2011). Besides, up to 100 nematode species from 50 genera, have been reported to be associated with soybean. Major nematode species limiting soybean production include the root-knot nematode *Meloidogyne* spp., cyst nematode *Heterodera glycines*, lesion nematode *Pratylenchus brachyurus*, and the reniform nematode *Rotylenchulus reniformis* (Lima et al., 2017).

The root-knot nematode (RKN) species *M. incognita, M. javanica*, and *M. arenaria* are the most common in the warm climatic regions and cause great losses in soybean crop yield (Da Conceição et al., 2009; Chaudhary et al., 2011; Tariq-Khan et al., 2017). There are different control methods against RKNs, such as chemical nematicides, biological control, crop rotation, and resistance cultivars (Abdelsalam et al., 2019; Fouda et al., 2020; Ghareeb et al., 2020a). Some of these chemical nematicides have been banned or their use has been restricted due to their undesirable negative impacts on the environment and human health (Alfy et al., 2020; Fouda et al., 2020). Therefore, the management of RKNs with biological control agents has been receiving increasing attention (Abd-Elgawad and Aboul-Eid, 2005; Abd-Elgawad and Kabeil, 2010). Pathogens can be divided into two main groups: first, pathogens in the biotrophic group stimulate the pathway of salicylic acid (SA). Accordingly, they activate the transcription of the non-expressor pathogen-related gene, which causes the signature gene of SA to induce the expression of PR1, PR2, and PR5. The accumulation of the PR1,2,3 gene expression will lead to systemically acquired resistance. The second group of pathogens comprises necrotrophic pathogens which stimulate the jasmonic acid pathway (JA), resulting in the activation of the key JA genes (PR3, PR4, and PR12) and finally the high accumulation of the PR3,4,12, proteins, leading to locally acquired resistance (LAR) (Ali et al., 2017; Ullah et al., 2019; Kou et al., 2021).

Nowadays, one important biological control tool is the use of suppression effects of cyanobacteria (blue-green algae) on plant-parasitic nematodes (Singh et al., 2011). Cyanobacteria have been reported to produce many metabolites, which have been identified as toxins, antibiotics, and protease inhibitors (Rastogi and Sinha, 2009; Schwarzenberger et al., 2010; Singh et al., 2011); some have been chemically characterized as neurotoxins causing the inhibition of acetylcholine esterase and/or functions such as acetylcholine. This is essentially because several cyanobacteria produce various biologically active compounds that could be utilized in the biological control of plant pathogens (Kulik, 1995; Kim, 2006). These bioactive compounds are biologically active molecules that, at low concentrations, affect a living organism, tissue, or cell beneficially or detrimentally (Armstrong et al., 2019). The extracted mixture of unsaturated fatty acids of *Oscillatoria redekei* exerts antimicrobial activity, which can be attributed to the ability of cyanobacteria to produce a large number and variety of bioactive chemical substances with a diverse range of biological activities and chemical structures that can affect many biochemical processes within cells. Many cyanobacteria species have yet to be studied for their nematicidal effects. Several studies have been conducted to investigate the nematicidal effects of cyanobacteria (Dutta et al., 2007; Chandel, 2009). Cyanobacterial extracts have siginificantly increased plant-parasitic nematode mortality and/or hatching inhibition following the inoculation of soil with endospores or extracts and exudates from cyanobacteria (Sharma et al., 2007). Similar to RKNs, egg masses and galls were also reduced after soil was mixed with cyanobacterial extract (Youssef and Ali, 1998; Khan and Park, 1999; Khan et al., 2005). Several cyanobacterial species are still under investigation to examine their effects on RKNs.

## Materials and Methods

### Sample Collection

#### Root-Knot Nematode Inoculum Preparation

For the inocula, *M. incognita* was extracted from a pure culture that was previously morphologically characterized and identified from adult female perennial patterns according to Branen et al. (2001), Shimizu (2003), Venugopal et al. (2005) and reared on tomato plants (*Lycopersicon esculentum* cv. Farida) grown in the greenhouse, watered, and fertilized as needed. The plants were uprooted after 45 days, and roots were gently washed and examined for nematode infection. Infected tomato roots were used to inoculate other tomato plants. Subculturing and maintenance were continuously carried out to achieve enough inoculum as second-stage juveniles (J2s) from soil, extracted using 1% of sodium hypochlorite solution for 2 min (Hussey, 1973) and incubated for 2 days at room temperature. Hatched J2s were used for analysis and Soybean greenhouse pot experiments.

#### Cyanobacterial Strain Isolation and Cultivation

A blue-green microalga (cyanobacterial sample) was isolated from freshwater bloom from New Nubariya, El Beheira Governorate, Egypt, at latitude 30°40′N and longitude 30°04′E. A 10 ml of the freshwater algal bloom sample was inoculated into a 100 ml BG-11 medium (Rippka et al., 1979) in a 250-ml conical flask and then incubated on a rotary shaker at 25 ± 2°C under white fluorescent light at 4,000-lux illumination, under a 12:12 h light/dark cycle, with constant aeration (Anderson et al., 2006). Every 3 days, the flasks were examined for algal growth and monitored using an optical microscope. Serial dilutions were prepared in BG-11 from flasks showing algal growth. Subcultures were made by inoculating 50 μl of algal solution onto Petri plates containing BG-11 solidified with 1.5% (w/v) of bacteriological agar. Furthermore, 50 μl aliquot of the same dilution were placed into wells of a 96-microtiter plate containing 200 μl of BG-11. Both Petri and microtiter plates were incubated at 27 ± 2°C under continuous illumination with white fluorescent light (4,000 lux) for 21 days. The purity of the algal culture was ensured by repeated plating and regular observation under a compound microscope (John et al., 2021).

### Identification of Cyanobacterial Strain

The cyanobacterial isolate was preliminarily identified based on its morphology (Annadotter et al., 2001) using a compound microscope (Olympus, BX40 microscope, Japan) and was confirmed using 16Sr RNA gene sequence analysis. Total genomic DNA was extracted from 10 ml of 21 days cyanobacterial pure culture by centrifugation at 8,000 × g for 15 min at 4°C using the bacterial DNA extraction protocol (Bornet and Branchard, 2004). In brief, the harvested cyanobacterial culture was resuspended in 50 μl of Tris-EDTA (TE) buffer at pH 7.4. Then, 750 μl of freshly prepared XS buffer (1% potassium ethyl xanthogenate; 100 mM Tris-HCl, pH 7.4; 20 mM EDTA, pH 8; 800 mM ammonium acetate; and 1% SDS) was added. The mixture was incubated in a water bath at 70°C for 2 h. After incubation, the tubes were vortexed for 30 s before being placed on ice for 30 min. DNA was purified successively using the 3 extraction solutions, namely, chloroform:isoamyl alcohol (24:1), phenol:chloroform: isoamyl alcohol (24:24:1), and chloroform:isoamyl alcohol (24:1). The purified DNA was dissolved in 30 μl TE buffer. Then, 5 μl of the extracted DNA was mixed with 2 μl loading dye and loaded onto 1.5% agarose gel to confirm the presence of DNA product.

#### 16S rRNA Amplification, Sequence, and Analysis

The 16S rRNA gene was amplified by using universal bacterial primers. The forward primer was 5′-AGAGTTTGATCMTGG CTCAG-3′ and the reverse primer was 3′-TACGGY ACCTTGTTACGACTT-5′ (Hamida et al., 2020). PCR amplification mixtures were prepared in 0.2-ml sterile PCR tubes by the addition of a final volume of 25 μl of the following reagents: 7.5 μl dH_2_O, 12.5 μl Master Mix (Itron biotechnology—Korea), 1 μl of the DNA template, 2 μl PCR primer (10 pmol/μl) (separately for each primer). DNA amplifications were carried out using a thermal cycler (Eppendorf, Germany) in the following configuration for 30 cycles: an initial denaturation of 95°C for 2 min, 34 cycles at 95°C for 40 s, annealing at 56°C for 1 min, extension at 72°C for 1 min, and final extension at 72°C for 10 min. Then, the amplification products were preserved at 4°C (Ferrer et al., 2001). PCR products were subjected to electrophoresis on 1% agarose gel by applying a 100 V current for 45 min, after which the gel was stained with ethidium bromide and screened under the Gel Documentation System. The PCR products were purified from the agarose gel using the PCR Clean Up Column Kit (Maxim Biotech Inc, United States). The PCR products were sequenced using a DNA Automated Sequencer. The sequencing was carried out with the same primers of amplification using HiSeq-2000 (Macrogene Scientific Services Company, Korea).

#### Phylogenetic Construction Based on the Obtained DNA Sequence of the 16S rRNA Gene

Phylogenetic analysis sequence similarity was analyzed using a BLAST search (https://blast.ncbi.nlm.nih.gov/Blast.cgi) after several alignments with sequences of closely and strongly related species. Comparative analysis was done utilizing CLUSTAL-W (https://www.genome.jp/tools-bin/Clustal W) in the Bioedit program. BLAST and Evolutionary Genetic Analysis version 7 (MEGA 7) software were used to assess the similarity of the phylogenetic tree to other cyanobacteria species, which was constructed using the neighbor-joining (NJ) method (Kumar et al., 2016). The other cyanobacteria which were used in the construction of the phylogenetic were selected based on their strong similarity (more than 96%) to our isolate when the DNA BLAST search was performed.

### Extract Preparation of *Oscillatoria* sp.

During the stationary phase of algal growth, ~21-day-old *Oscillatoria* sp. biomass was harvested by centrifugation at 4,000 × g for 15 min, the aqueous phase was discarded, and the pellets were dried in an oven at 45 ± 2°C until a constant weight was achieved, after which they were subjected to extraction. Then, 10 g/L(S%) of *Oscillatoria* dry weight was extracted separately either by methanol solvent or by water at 55 ± 5°C by immersing the *Oscillatoria* powder in two different flasks and using an ultrasonic micro-tip probe of 100 W for 15 min for extraction; then, the extract was incubated at 37°C with constant shaking for 1 week at 150 rpm to confirm all cell content and thawed out, which was broken down and blasted by sonication. The methanolic extract was concentrated to dryness in a rotary evaporator at 35°C ± 2. In the case of the water extract, centrifugation at 8,000 × g for 20 min was carried out. The combined supernatant was evaporated to dryness at 40°C using an oven for 7–10 days or until complete dryness was observed. Finally, the two dried extracts were collected and stored in labeled sterile vials in pre-weighed test tubes and then preserved at 4°C (Chauhan and Johnson, 2010).

#### Qualitative Analysis of Common Components Existing in Oscillatoria Cyanobacteria Extract

The phytochemical screening of *Oscillatoria* extract was conducted using standard methods as described below. Phytochemical screening was performed to identify the major natural chemical compounds, such as phenols, tannins, flavonoids, glycosides, saponins, proteins, and amino acids. The analyses determined the presence/absence of these compounds in the algal extract.

#### Detection of Phenolic Compounds

The total phenolic concentration was measured using the Folin–Ciocalteau method (Yildiz et al., 2011). First, 100 μl of algal extract with an extract concentration of 1,000 μg/ml of water was mixed with 2.0 ml of 2% Na_2_CO_3_ and allowed to stand for 2 min at room temperature. Then, 100 μl of 50% Folin–Ciocalteau phenol reagent was added and the mixture was incubated for 30 min at room temperature in the dark, after which the absorbance was measured at 720 nm using a spectrophotometer (Milton Roy Spectronic 1201) (Taga et al., 1984).

#### Detection of Tannins

Total tannin content was assayed according to the method of Atanassova and Christova-Bagdassarian (2009) with a slight modification. Briefly, a 50 μl aliquot of *Oscillatoria* extract was mixed with 1.5 ml of 4% vanillin (prepared with methanol); then, 750 μl of concentrated HCl was added. The solution was shaken and then left to stand for 20 min at room temperature in the dark. Absorbance was measured against a blank at 500 nm.

#### Detection of Flavonoids

Total flavonoid content was determined according to the method of Martins et al. (2011); and Shi et al. (2012). A 250 μl aliquot of *Oscillatoria* extract was mixed with 1.25 ml of double-distilled (dd) water and 75 μl of 5% NaNO_2_ solution. After 6 min, 150 μl of 10% AlCl_3_.H_2_O solution was added. After 5 min, 0.5 ml of 1 M NaOH solution was added and then the total volume was adjusted to 2.5 ml with water. Following the mixing of the solution, the absorbance against a blank was measured at 510 nm.

#### Determination of Glycosides

For glycoside identification, 3 ml of chloroform and 10% ammonium solution wereadded to 2 ml of the *Oscillatoria* extract. The appearance of a pink color indicated the presence of glycosides (Cuellar-Bermudez et al., 2015).

#### Determination of Saponins

Two grams of the dried *Oscillatoria* extract were boiled in 20 ml of dH_2_O in a water bath and then filtered. A 10 ml of the filtrate was mixed with 5 ml of dH_2_O and shaken vigorously to achieve a stable, persistent froth. The frothing solution was mixed with 3 drops of olive oil and shaken vigorously until the formation of an emulsion was observed (Abhaykumar, 2018).

### Nematode Mortality Bioassays

To determine the effect of the algal extract on *M. incognita* juveniles, the mortality rates were examined under different concentrations of the algal extract [i.e., 100 (S%), 50 (S/2), and 25 (S/3)% concentrations]. The bioassay was performed in a sterile 6-well cell culture plate, and each treatment was represented by ~50 freshly hatched J2s per well compared with the control treatment containing dH_2_O, with five replicates each, and the experiment was repeated twice. The plates containing nematode juvenile solution and algal extract were incubated at 27 ± 2°C, and the mortality of J2s was determined after 6-, 12-, 24-, and 72-h intervals. After treatment, juveniles were transferred to plain water. Larvae were considered alive if they moved in a winding pattern (El-Rokiek and El-Nagdi, 2011), but they were considered dead if they did not regain movement after being transferred to plain water and when probed with a fine needle (Aissani et al., 2015). The percentage of juvenile mortality was calculated by the following formula (Grewal, 2000; El-Elimat et al., 2012).


(1) $$\begin{array}{l} \;Mortality\;\%\;M\;\%\;=[(Total\;numberof\;J2s\;in\;control-\\ No.\;of\;alive\;J2s\;intreatment)/No.\;of\;Total\;J2s\;in\;control]\times 100 \end{array}$$

### Egg Hatchability Assay

The bioassay to assess nematodes' egg hatchability was performed using a sterile 6-well cell culture plate (SPL Life Sciences Co., Ltd. Korea), containing ~100 eggs in each well. Three concentrations (100%, 50%, and 25%) of the algal extract were assessed, in addition to the aqueous crude extract with 100% and Oxamyl (Vydate SL 24%), 2-(dimethylamino)-*N*-[[(methylamino) carbonyl] oxy]-2-oxoethanimidothioate, obtained from Shoura Chemicals on Alexandria Desert Road, Egypt, at the recommended rate (0.026 mg Oxamyl/10 ml). The plates containing dH_2_O served as controls. Five replicates were made, and each experiment was repeated at least two times. All the treatment plates were incubated at 27 ± 2°C, and the hatching was recorded after 3, 7, and 15 days. After each count, the eggs were washed with 1 ml of dH_2_O in their respective plates and transferred to plates with fresh particles of the same concentration. The percentage of egg hatch inhibition was calculated according to Grewal (2000) and El-Elimat et al. (2012).


(2) $$\text{Egg hatchability =}\frac{Total\;eggincontrol-Egg\;intreatement}{Egg\;hatching\;in\;control}\times 100\text{​​​​}$$

### Greenhouse Experiment

The *Oscillatoria* extracts were evaluated to examine their efficacies against juveniles of *M. incognita* in soybean (*Glycine max* L. cv. Giza 111) transplants of similar age and size (two-leaf stage), 1 week before transplanting. Single transplantation was performed in a plastic pot (20-cm diameter and 15-cm depth filled with 3 kg mixture of sand:clay 1:1, v:v) and plants were watered daily (where needed) to ensure the establishment of the organisms in the soil. Each pot (one uniform soybean seedling) was inoculated with 4,000 second-stage juveniles (J2s)/pot week after the time of transplantation by pouring the nematode suspension into holes made at a depth of 5–7 cm below the soil surface, around the base of the plants. Five pots of soybean seedlings left without inoculated nematodes served as control. All pots were arranged in a completely randomized block design on the bench in a greenhouse that averaged 22 ± 5°C. The experimental treatments, each with five replicates, were the following: (1) non-inoculated control; (2) untreated control inoculated with *M. incognita*; (3) treatment inoculated with *M. incognita* and treated with Oxamyl 24% SL at the recommended rate (5 ml/l) were applied as a soil drench in 150 ml H_2_O/pot; and (4) treatment inoculated with *M. incognita* and treated with *Oscillatoria* extract (10 g of *Oscillatoria* powder was soaked in 100 ml). All treatments were applied directly after 24 h of nematodes inoculation. A total of 45 days after inoculation, the soybean plants were uprooted gently and washed free of adhering soil. Parameters including length, fresh and dry weight of shoots, and fresh and dry weight of the root system were determined and recorded. In addition, chlorophyll content in leaves was determined following Lichtenthaler et al. (1996). Infected soybean plant roots were examined for the number of galls, females, and egg masses per root system and recorded after roots were stained for 15–20 min in an aqueous solution of Phloxine B stain (0.15 g/l) (Fan et al., 2020). The second-stage juveniles (J2s) per 250 g soil were extracted from the soil according to Cobb's sieving and decanting method by using sieves (60-mesh and 325-mesh).

### Effect of two Extracts of *Oscillatoria* on Soybean Defense Gene Expression

#### Extraction of Total RNA From Soybean Plant Tissues

RNA isolation from plant roots (healthy and infected) was carried out according to the protocol of the TRIzol reagent (Wang et al., 2009). Soybean plant tissues were ground to a fine powder in liquid nitrogen with a pre-cooled pestle and mortar and then placed separately into a 50-ml plastic Eppendorf centrifuge tube. For 0.1 g samples, 1 ml extraction buffer [TRIzol reagent: 38% phenol (USB Cooperation, Cleveland, OH, United States) was equilibrated to pH 4.0 with Tris-HCl buffer; 0.8 M guanidine thiocyanate; 0.4 M ammonium thiocyanate; 0.1 M sodium acetate (pH 5.0); 5% glycerol] was added and mixed well. Samples were incubated for 15 min at −20. After incubation, 0.2 ml chloroform was added for each 1 ml extraction buffer and tubes were shaken vigorously with a vortex for 15 s. Tubes were incubated at room temperature for 5 min and then centrifuged at 10,000 × g for 15 min at 4°C. The aqueous layer was carefully transferred into a clean Eppendorf centrifuge tube and then 0.5 ml of isopropanol was added for every 1 ml of extraction buffer.

Tubes were covered and mixed by gentle inversion and then left at room temperature for 10 min; tubes were centrifuged (10,000 × g; 4°C; 10 min) and the supernatant was discarded. Pellets were washed in 75% ice-cold ethanol and centrifuged (5,000 × g; 4°C; 5 min) and the supernatant was discarded; pellets were dried for 10 min. For each gram of tissue, 50 μl Diethyl pyrocarbonate (DEPC) from (Sigma, United States) treated H_2_O was added to dissolve the pellets, and tubes were kept at 4°C overnight and then re-centrifuged (5,000 × g; 4°C; 10 min). The supernatant was discarded and RNA pellets were dissolved in RNase-free water. Total RNA concentration was determined by measuring absorbance at 260 and 280 nm. Samples were stored at −80°C until further use.

#### Synthesis of cDNA

For the first strand, cDNA was synthesized using reverse transcriptase (Fermentas, United States) and buffer (5X) [50 mM Tris-HCl (pH 8.3 at 25 OC), 250 mM KCl and 20 mM MgCl_2_ and 50 mM DTT] in the presence of a random hexamer primer (Promega, United States) and 5 μl of RNA was added to [10 μl (5x) RT—buffer, 5 μl (25 mM) dNTPs, 5 μl of primer, 0.5 μl (20 U/ μl) of RT—enzyme, 24.5 μl H_2_O]. The mixture was incubated at 37°C for 60 min and then at 70°C for 10 min (for enzyme inactivation) followed by storage at 4°C until use (Sambrook and Russell, 2001).

#### Differential Display - PCR for the Treated and Untreated Soybean Plants

Up- and downregulation of the four genes involved in the plant defense system were tested by DD-PCR in plant roots, 7 and 15 days after treatment with the *Oscillatoria* extracts, and commercial nematicides were compared with untreated controls. At first, 4 genes belonging to the PR-1, PR-2, PR-5, and PR-12 gene families were tested. DD-PCR reactions were prepared for each 25 μl PCR reaction by mixing 12.5 μl PCR reaction (Green Hot Start PCR Master Mix (2X), Invitrogen Co.), 2 μl (40 pmol/ μl) of each primer, pathogenesis-related protein (PR1), CTGGAGCACGAAGCTGCAG,β-1,3-glucanase(PR2), CCGATAACCATGGCTTCTTCTTCTCTGCAGTC-3′, Thaumatin-like protein (PR5), CGCGTCCTAATCTAAGGGCAG and Defensin gene-1 (PR12), 5'-CACAGAAGTTGTGCGAGAGG-3′, 3 μl cDNA, and 6.5 μl dH_2_O freeRNase. The program was designed as follows: for the first cycle, samples were initially incubated for 5 min at 94°C, followed by 40 cycles at 94°C for 30 s, 42°C for 1 min, and 72 for 1 min. An additional extension period at 72°C was programmed for 5 min. The resulting PCR products were analyzed with 0.5 × TBE as a running buffer in 1.5% agarose gels. Electrophoresis was performed at 80 V for 90 min and then the gel was stained in 0.5 μg/cm^3^ (w/v) ethidium bromide solution and destained in deionized water. Finally, DNA fragments were visualized under UV light and photographed using a Gel Documentation System (Zhao et al., 2020).

### Statistical Analysis

Data collected *in vitro* and during pot experiments were processed using spreadsheet software Microsoft Excel and analyzed by two-way analysis of variance (treatments and times) using SAS software version 9.4 (SAS Institute Inc., Cary, NC, United States) to determine significant differences between mean values at the probability level of 0.05.

## Results

### Identification of the Isolated Microalga

**Figure 1:**
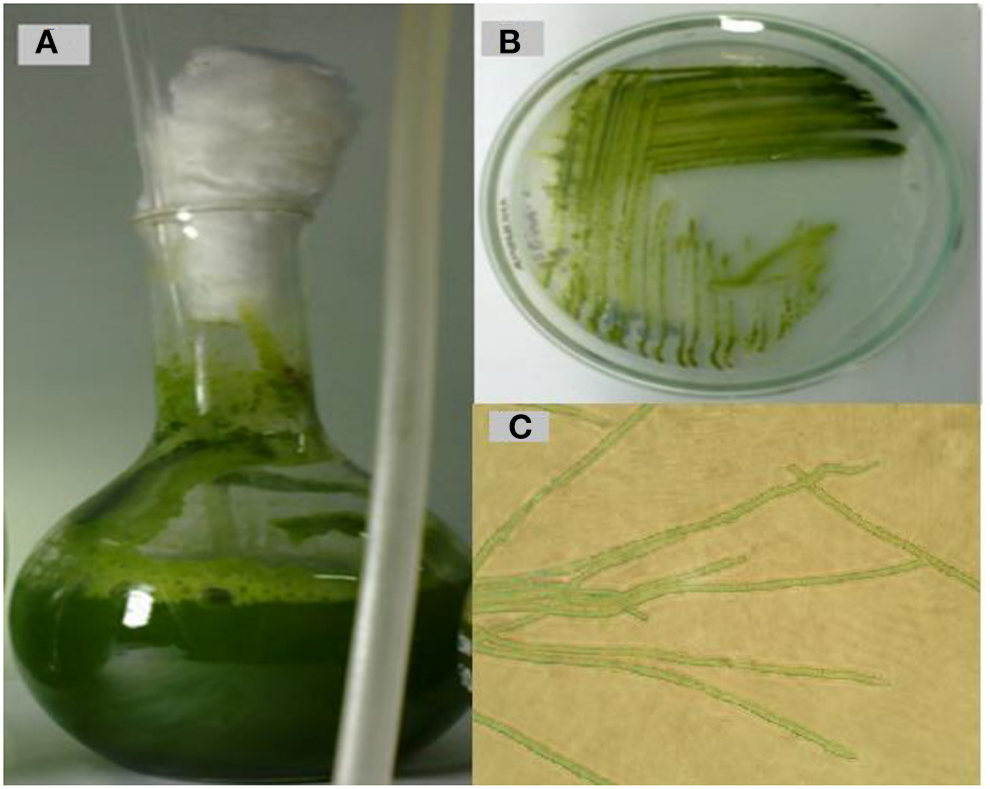
Cyanobacterium isolation, cultivation, and purification. **(A)** Photo showing the growth after 21 days at 25 °C on BG11 liquid media. **(B)** Culture on solid BG-11 medium. **(C)** Magnified light micrograph of *Oscillatoria* sp. (40 ×).

Cyanobacteria are considered highly important because they produce a huge number (2,000–8,000) of bioactive compounds as secondary metabolites (Martins et al., 2011). For instance, the isolated cyanobacteria were subjected to complete morphological and molecular identification. The collected cyanobacteria were cultured, and a pure isolate was selected based on purity and growth rate. Microscopic examination of the selected algal isolate revealed the presence of filamentous organisms; additionally, it was found to be non-heterocystous cyanobacteria because the cells composing the trichome were disc-shaped and not separated by a deep constriction. Cells did not contain gas vacuoles and were either linear or slightly curved. Cell size ranged from 1.3 to 2.2 μm in width and 4–12 μm in height depending on the growth stage (Figures 1A–C). As shown in Figure 1C, cell walls become noticeably narrower with a narrow tip.

**Figure 2:**
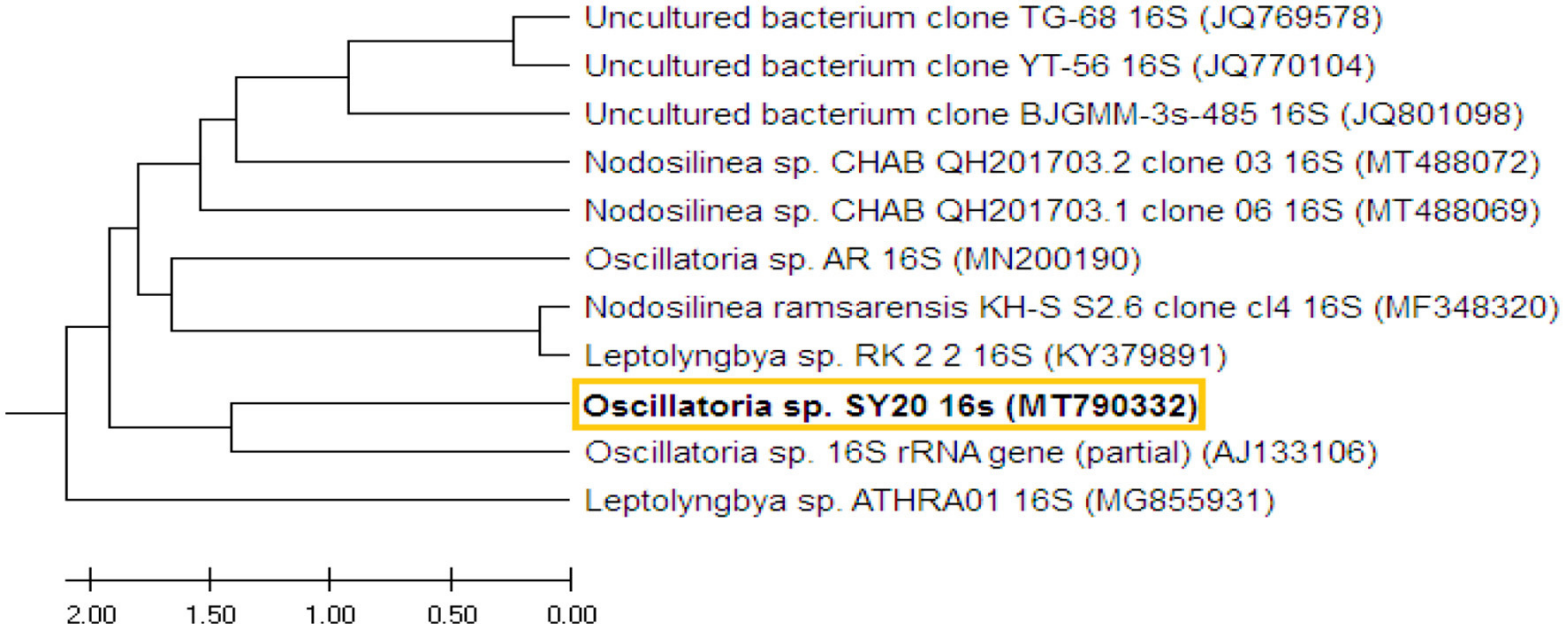
The phylogenetic tree shows the DNA sequence of the 16S region of *Oscillatoria* and the DNA nucleotide sequences of 10 other 16S rRNA genes of 10 different cyanobacterial strains. The phylogenetic tree was constructed based on the DNA sequences of the selected strains obtained from GenBank.

The 16S rRNA gene was amplified from the selected pure isolate and sequenced (670 bp). The DNA sequence was submitted to GenBank, named with *Oscillatoria* sp. SY20, under the accession no. MT790332.1. BLAST results showed that the obtained *Oscillatoria* sp. was highly similar (99%) to *Oscillatoria* sp. (accession no. AJ133106). The tree with the highest log likelihood (−153.4067) is shown in the Supplementary Materials. The analysis involved 11 DNA nucleotide sequences of 11 different *Oscillatoria* sp. strains (Figure 2).

### Growth Rate and Biomass Yield of *Oscillatoria* sp.

**Figure 3:**
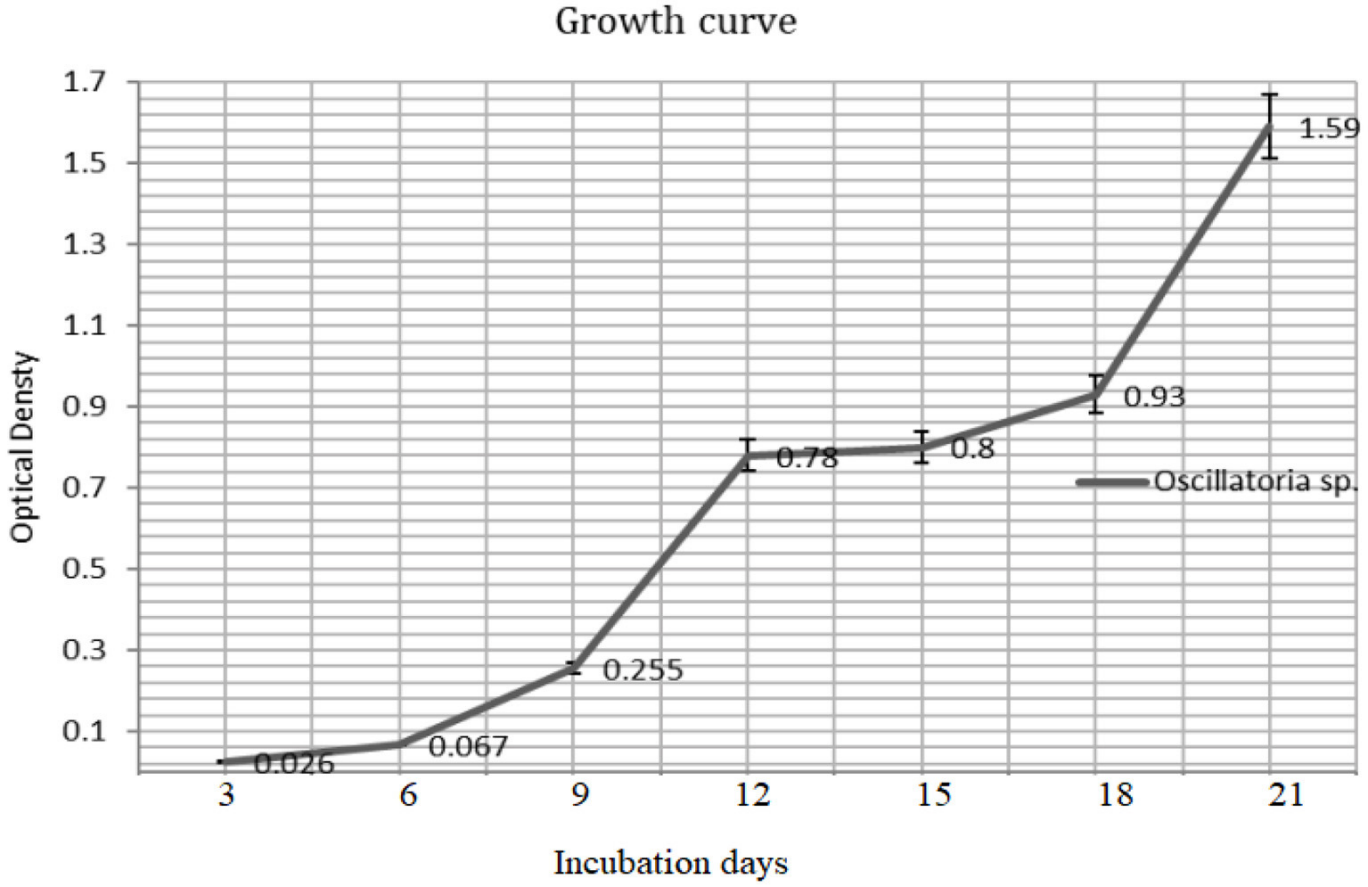
The growth rate of *Oscillatoria* sp (O.D_680_) grown on BG-11 liquid culture for 21 days.

**Figure 4:**
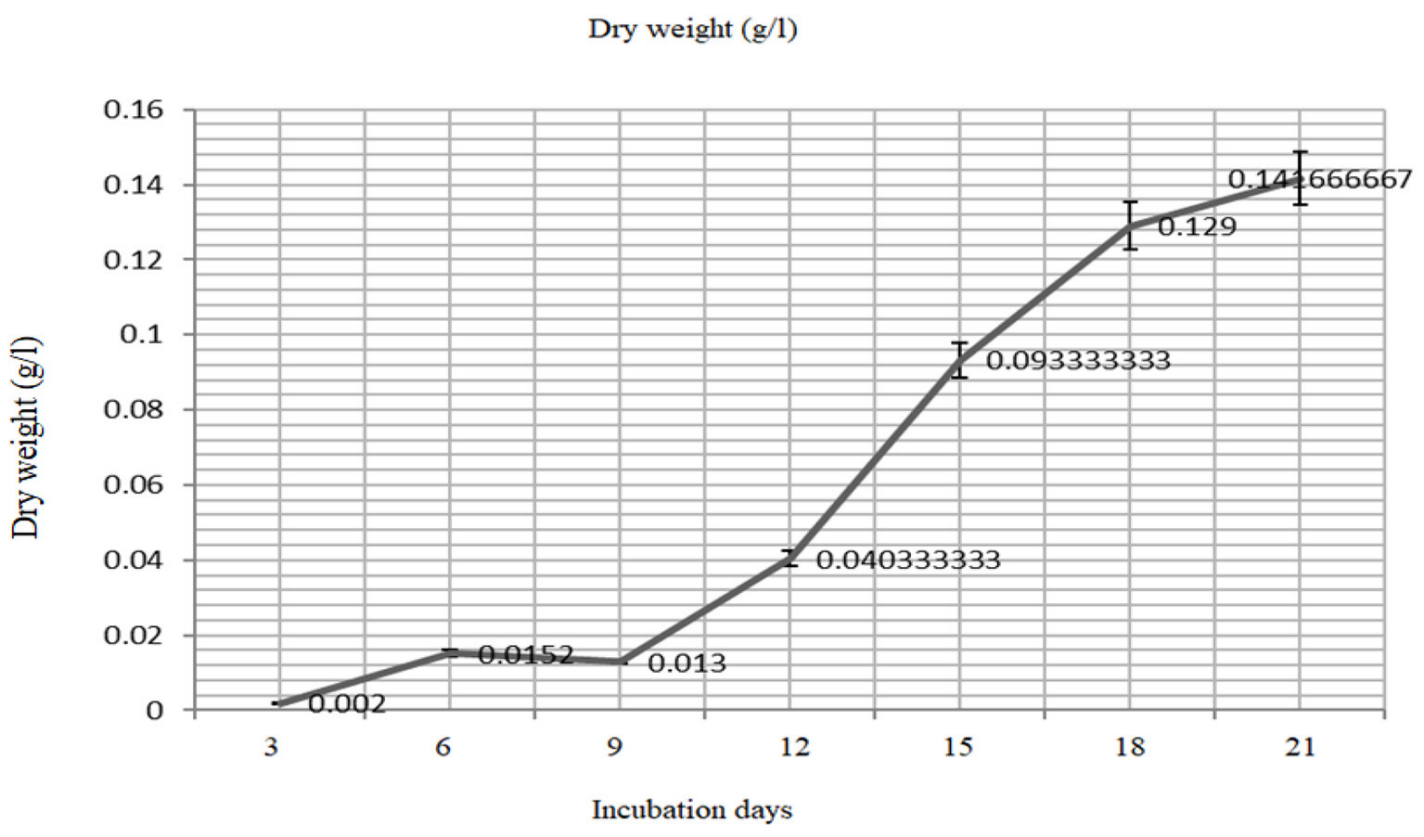
The mass production (dry weight) of the *Oscillatoria* sp. grown on BG- medium culture 21 days.

The biomass yield reaches its peak after 21 days (680 nm) of inoculation and the same observation was recorded for the resulted dry weight of 1.59 ± 0.03 g/l after 21 days (Figures 3, 4).

### Qualitative Analysis of Phytochemical Contents of the *Oscillatoria* sp. Extract

Chemical analysis of plants obtained from *Oscillatoria* sp. The presence of phenolic compounds, tannins, flavonoids, glycosides, and saponins was detected in the extract, as shown in Table 1, which were the most dominant phytochemicals, phenol, tannins, and flavonoids, respectively.

**Table 1:** Qualitative analyses of phytochemicals of the extract of *Oscillatoria* sp.

**No**.	**Phytochemical components**	***Oscillatoria* extract**
1	Phenols	++
2	Tannins	++
3	Flavonoids	++
4	Glycosides	+
5	Saponins	+

*++ high optical density, + moderate optical density*.

### Bioassay Evaluation of the Effects of *Oscillatoria* Extracts on *Meloidogyne incognita* Under Laboratory Conditions

**Figure 5:**
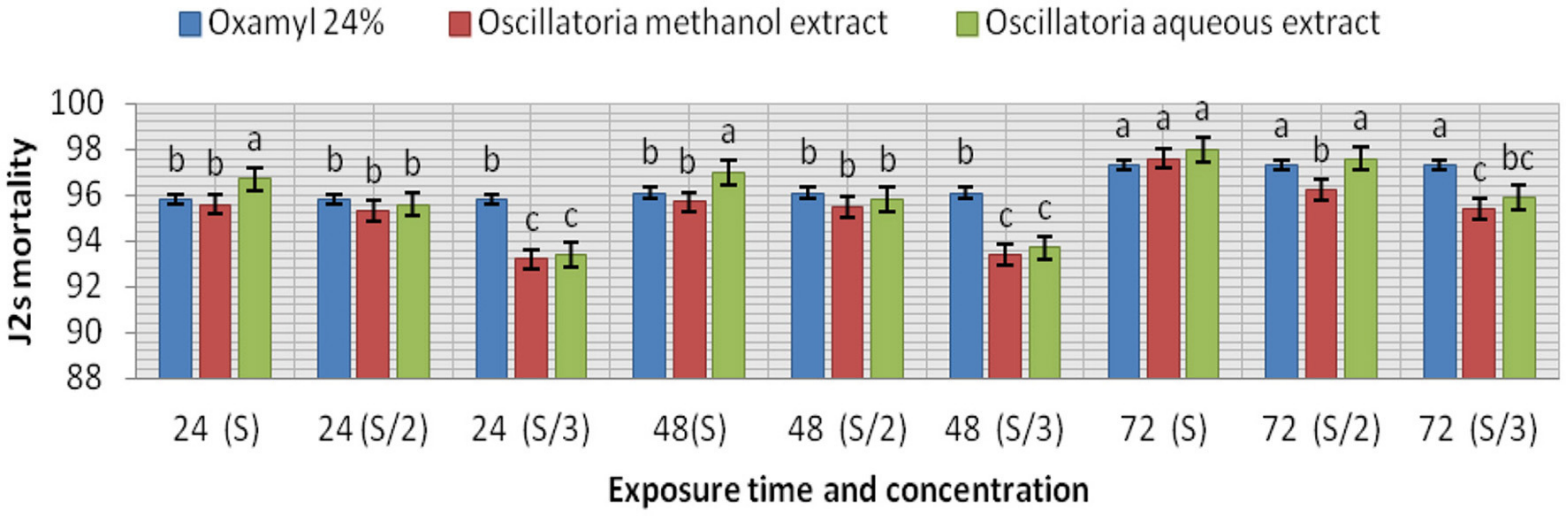
Evaluation of the nematicidal effects of aqueous and methanol *Oscillatoria* extracts compared with Oxamyl on J2 of *M. incognita* after 24, 48, and 72 h, reduction % (R).

**Figure 6:**
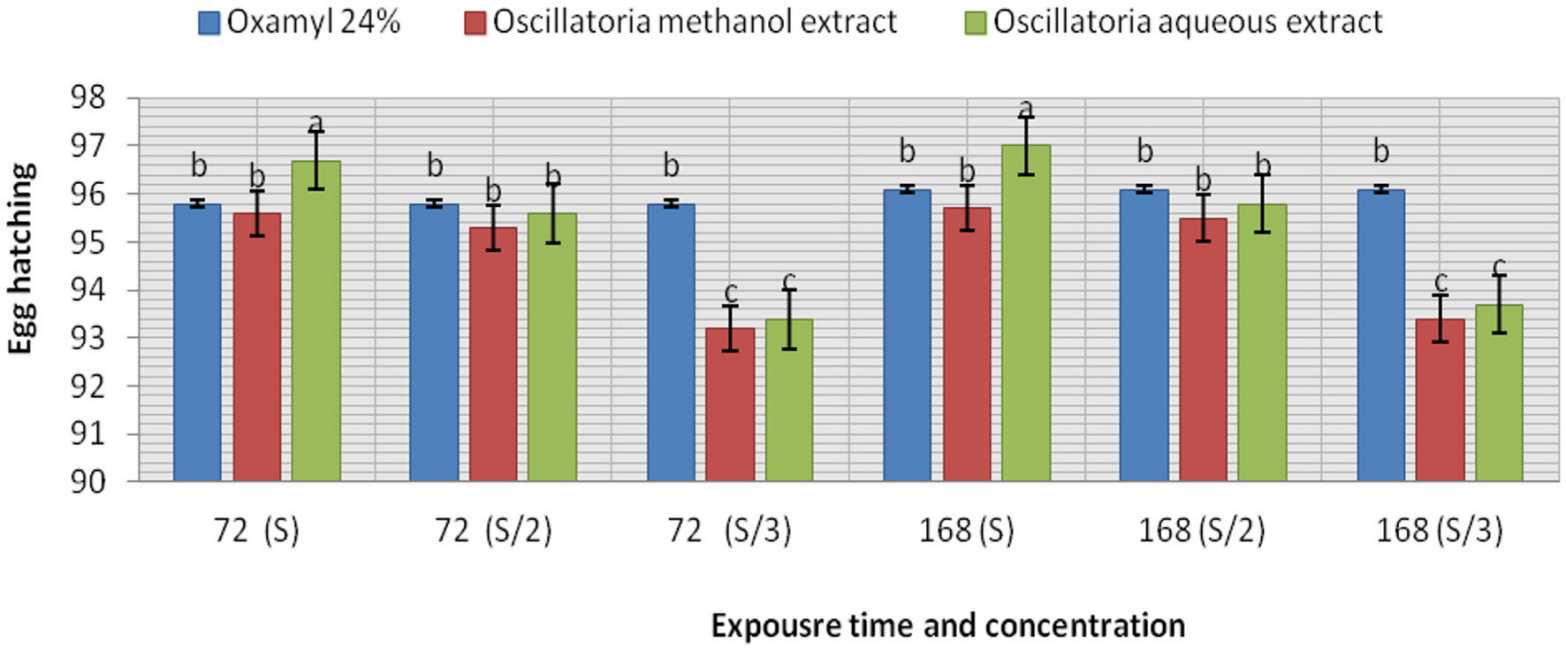
Evaluation of the nematicidal effects of aqueous and methanol *Oscillatoria* extracts and Oxamyl on J2s of root-knot nematode *M. incognita* egg hatchability after 72 and 168 h, reduction % (R).

The extracts (methanol and hot water) of the microalga *Oscillatoria* sp. were tested for nematicidal activity against J2s of root-knot mortality and egg hatching (Figures 5, 6). It was observed that both the mortality percentage and the reduction percentage of egg hatching were increased by increasing the exposure time. The mortality of *M. incognita* juveniles was evaluated using hot water extracts (S and S/2) followed by extracts (S) with 98, 97.6, and 97.6% compared to Oxamyl 24% with 97.3%, respectively. Consequently, according to the data presented in Figure 6 and after 72 h exposure, it was observed that the greatest reduction in the egg hatchability of *M. incognita* (96.7%) was obtained with the hot water extract (S), followed by Oxamyl 24%, with a reduction percentage of 95.8%. After 16 h of exposure, the percentage reduction in egg hatchability increased (97% with S and 96.1% Oxamyl 24%, respectively).

### Nematicidal Activities of *Oscillatoria* Extract Against Root-Knot Nematode *in vivo*

**Figure 7:**
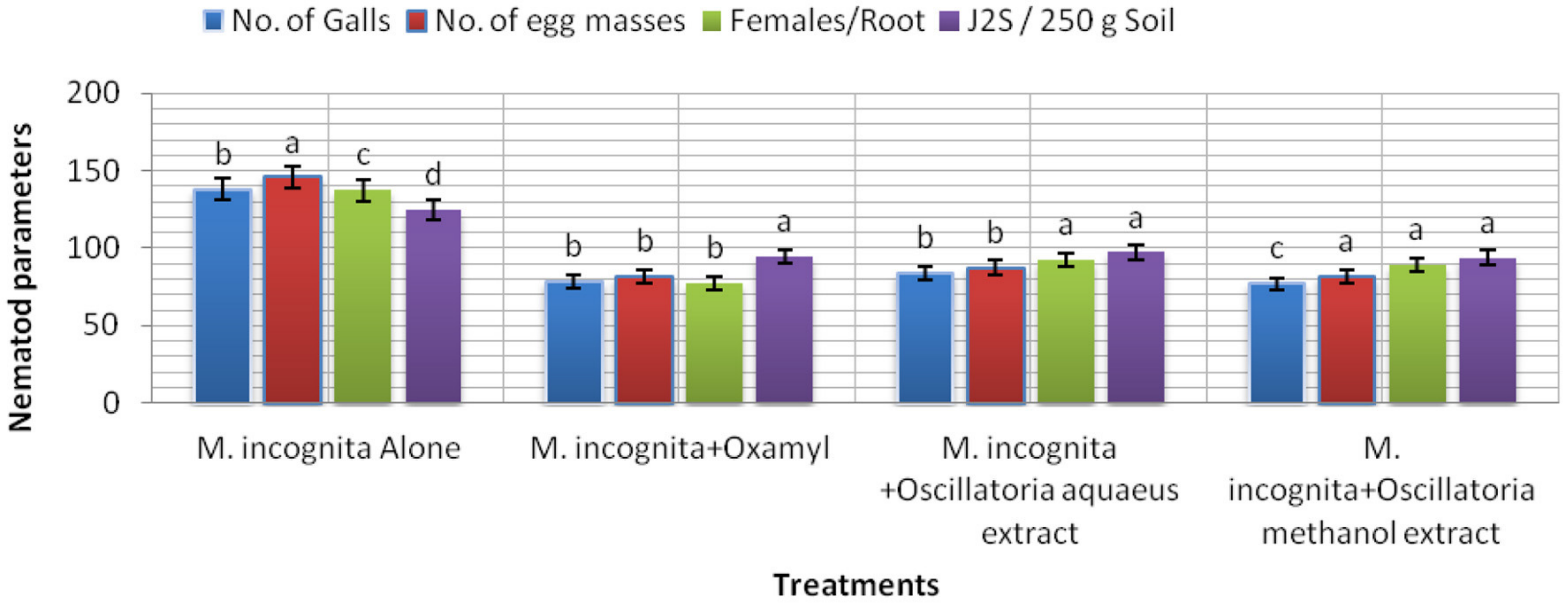
The effect of *Oscillatoria* sp. methanol and hot water extracts on the number of nematode galls, egg masses, females/root system, and the number of second-stage juveniles (J2S)/250 g soil in soybean plants infected with *M. incognita* (Mj) after 60 days cultivation.

The results listed in Figure 7 clarify the notion that all treatments (Mi+ *Oscillatoria* hot water extract with concentration (S), Mi + *Oscillatoria* methanol extract with concentration (S), and the nematicide, Oxamyl) achieved a significant reduction and reduced the number of galls and EM of *M. incognita* by 84.1–77% and 87.5–81.8% compared with the control, Oxamyl, with values of 78.3% and 81.7%, respectively. Meanwhile, the number of females was reduced, with reductions of 92.2, 89, and 77.3%, respectively. However, data indicated that the numbers of J2s/250 g soil were reduced with all treatments, with reductions of 93.7–97.3% and 94.6%.

**Figure 8:**
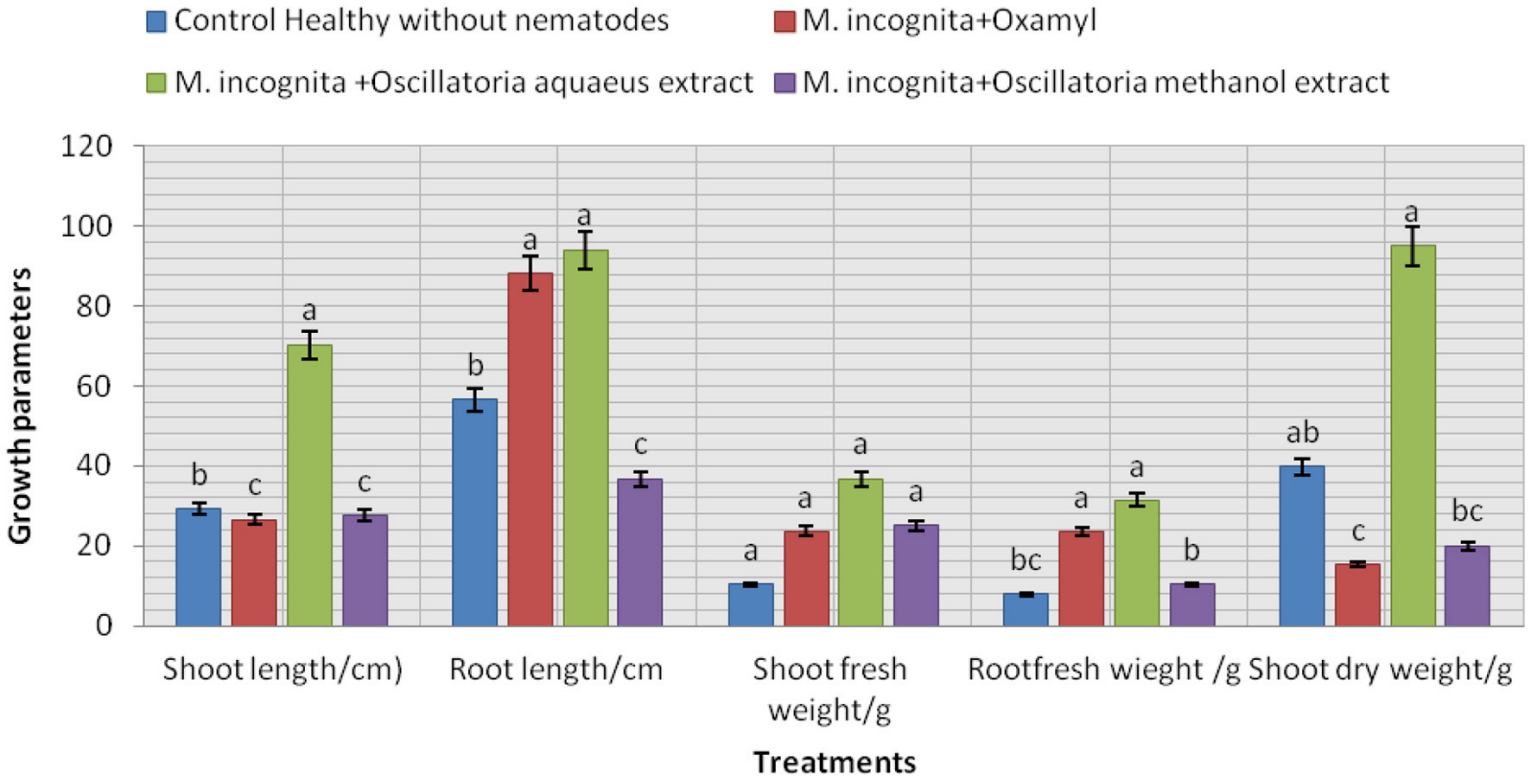
Evaluation of the nematicidal effects of *Oscillatoria* sp. methanol and hot water extracts on soybean growth parameters, increase (%) as well as chlorophyll content.

The data presented in Figure 8 show that the influence of the tested treatment caused a remarkable increment in soybean plant growth to a certain extent. It is noticeable that all plants infected with nematodes and treated with the cyanobacterial extract (Mi+ Oscillatoria hot water extract with concentration (S), Mi + *Oscillatoria* methanol extract with concentration (S), and the nematicide, Oxamyl), compared with the control (plant infected with nematode), showed improved soybean growth parameters since the largest percentage increase in shoot and root length was recorded at 70.3 and 94.1% with Mi+ *Oscillatoria* hot water extract with concentration (S), respectively. Likewise, the weight of fresh roots and roots was increased by 36 and 31.4% increase, respectively, and the shoot dry weight was increased by 95.5% when compared with the nematicide Oxamyl, with values of 26.6, 88.2, 23.6, 23.5, and 15.38%, respectively. Moreover, all treatments caused significant enhancements in chlorophyll content, with values of 1.42, 1.3, and 1.3 b over the positive control (healthy) with 0.872 / U (mg/mg).

### *Oscillatoria* Extracts Activate the Immune Response of Soybean Plants

**Figure 9:**
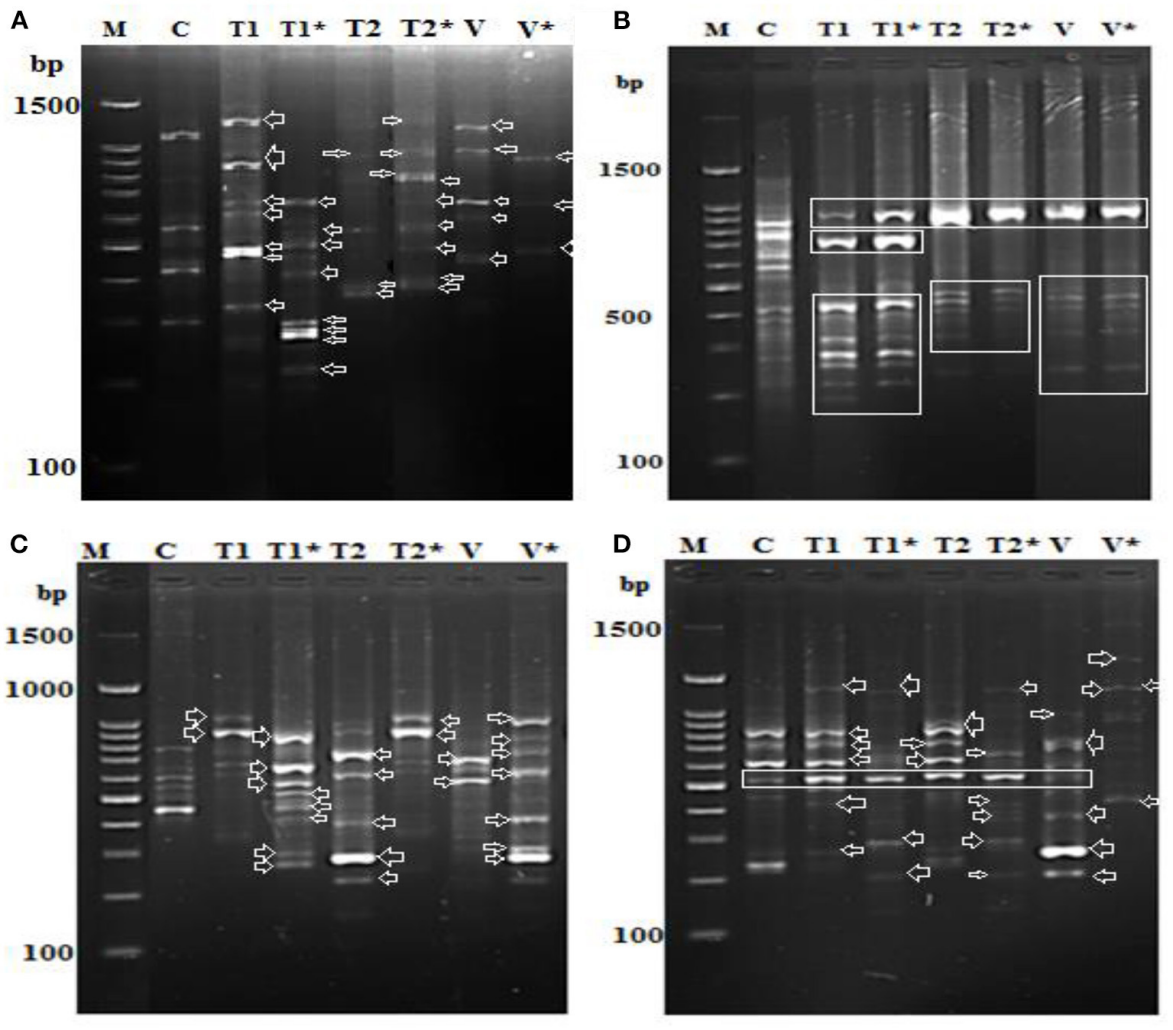
Differential display-PCR for soybean plants tested using primer **(A)** PR-1, **(B)** PR-3, **(C)** PR-5, and **(D)** PR-12 showing the polymorphic and monomorphic bands M: 1,500 kbp DNA marker, **(C)**: pooling of healthy, un-infected, and untreated (control) plants, (T1): pool of successful treatments with *Oscillatoria* hot water extract after 7 days and T1* after 21 days. **(D)**: T2 pool of successful treatments with *Oscillatoria* methanol extract as nematicide after 7 days and T2* after 21 days. (V): plants with nematode + Oxamyl after 7 days and V* after 21 days.

We found alterations in the PR-1, PR-2, PR-5, and PR 12 genes together with the downregulation of two genes involved in the defense system (Figures 9A–D). In addition, alterations in the expression of genes involved in PR5 and PR15 were observed, and numerous common bands between control (healthy) and infected soybean plants treated with *Oscillatoria* extracts were discovered. On the contrary, several bands were observed in the infected plants that were absent in the control and *vice versa*. Results generated by the gene encoding endo-1,3-beta-glucanase showed only one upregulated band with a molecular size of 550 bp at both time points [(T1) after 7 days and (T1^*^) after 21 days day after infection with *Oscillatoria* hot water extract] compared with the control at the same time point; on the contrary, this band appeared to be downregulated with Oxamyl at both time points when compared with the control (Figure 9A). Meanwhile, the differential expression results with the gene encoding the cell wall–modifying xylose demonstrated that four sharp bands with treatment T1, T1^*^, T2, and V (Oxamyl after 7 days at a molecular size of 1.5 k and, noticeably, this band disappeared with T2^*^ and V^*^ (Oxamyl after 21 days) compared with the control plant, appearing to be upregulated with a unique band at 200-bp molecular size with *Oscillatoria* hot water extract after 7 days following infection, as revealed in Figure 9B. In addition, gene PR-5 demonstrated the same sharp and unique band at both time points with all treatments, including the control treatment (Figure 9C).

Finally, the soybean plants treated with hot water extracts of *Oscillatoria* showed the presence of seven different upregulated bands in comparison with the control. However, at least five bands out of the seven were downregulated when the experiment reached 21 days following treatment (T1^*^). We assumed that the PR-12-related genes (mRNA) could be degraded or suppressed over time. On the contrary, only two bands were upregulated in the methanol extract of Oscillatoria (T2^*^) after 21 days following inoculation when compared with T2 after 7 days; it might be that this type of treatment prolonged the life of the mRNA of the PR-12-related gene. The same observation was made for the treatment with Oxamyl after 21 days following inoculation compared with Oxamyl after 7 days, demonstrated by the two upregulated bands shown in Figure 9D.

## Discussion

The results of the present study undoubtedly indicate that the extract of the cyanobacterial *Oscillatoria* sp. is a potential source of bionematicides against root-knot nematodes (*M. incognita*). The results indicate that aqueous and methanol cyanobacterial extracts of *Oscillatoria* possess numerous chemical ingredients, namely, alkaloids, flavonoids, phenols, tannins, and saponins. Phytochemical estimations have also shown that cyanobacteria are rich in alkaloids, phenols, terpenoids, flavonoids, and others, and exert an extraordinary amount of nematicidal activity (Okajima et al., 2008; Morone et al., 2019). It might be possible that the maximum inhibition of egg hatching and mortality in second-stage juveniles of *M. incognita* may be due to the presence of these phytochemicals in aqueous and methanol extracts of *Oscillatoria* sp.

Phytochemical compounds are naturally found in plants, and they perform many enzymatic reactions inside the plant cells. Among these compounds, flavonoid, phenolic, and quinone compounds in the extract tested in this investigation were shown to exert anti-nematode activity. These results agree with the results obtained by Thornalley et al. (2011) and Garcia-Bustos et al. (2019), who postulated that these compounds carry a strong anti-nematode capability, especially when they were tested against a *Caenorhabditis elegans* model nematode. Moreover, terpenoids also showed nematicidal activity when they were tested against the nematode *Haemonchus contortus* (Garcia-Bustos et al., 2019). In addition, it was reported that saponins, glycosides, and quinones exert phytocytotoxic activity and affect the interference of DNA and RNA replication inside nematode cells treated with the plant extract (Mahmud, 2014). Moreover, alkaloids, which are considered a major component of some plant extracts, showed strong activity against the larval stages of *H. contortus* and inhibited egg hatching and larval feeding and movement (Klein-Júnior et al., 2016; Nikmaram et al., 2018). The same observation was recorded for tannins, which were found to affect the L3 stage of the nematode Juan strain of *H. contortus* and reduced its egg hatching (Engström et al., 2016).

From the data presented in this investigation, it is evident that the application of the hot water extract of *Oscillatoria* cyanobacteria certainly achieved better results than either methanol extract or chemical nematicides. The algal extract not only controlled nematodes either in laboratory application or in the greenhouse experiment but also improved the growth parameters of the treated soybean plants with 96.7, 97, and 98 larvae (J2s) mortality (M) % with S concentration after 24, 48, and 72 h from exposure time and 96.7–97 egg hatching % after 72 h and 1 week after exposure time with the same concentration in a laboratory experiment. Therefore, hot water extracts significantly decreased the number of galls in soybean root, egg masses, and female/root with 84.1, 87.5, 92.2% and decrease the % of J2s/250 g soil by 93.7%. Root, shoot lengths, dry weight, number of pods/plant, and the chlorophyll content of soybean treated with Oscillatoria water extract were significantly higher compared with control with 70.3, 94.1, 95.5, and 2.02% increase. These findings are in contrast with those reported by Khan et al. (2015), who observed that the methanol extract was more effective as compared to a water extract and the filtrate of blue-green alga *M. vaginatus* culture killed the juveniles of *Meloidogyne incognita*; the increase in exposure period and concentration increased this effect. Moreover, Biondi et al. (2004) found that the nematicidal efficacy of methanolic extracts of the cyanobacterial strain was high when compared with results obtained with water extracts, and 50% concentrations of the methanolic extract slowed the nematode life cycle. The aqueous extracts of 10 species of cyanobacteria, namely, *Synechococcus nodules (*Pringsheim*)* Kotare*, Anabaena fertilissima, Aulosira pseudoramosa Bharadwaja, Micochaete* sp*., Nostoccommune Vaucher ex Bornet et Flahault, Oscillatoria fremyii* J., *Phormidium molle Kutzing ex* Gomont*, Tolypothrix phyllophila* West et G.S. West, and *Westiellopsis prolifica* Janet caused 57–69% inactivation of J2s of *M. incognita* in 24 h. Lyngbya sp., *O. fremyii, P. mole*, and *W. prolifica* caused greater inactivation compared to others (Liu et al., 2006).

To study the effect of cyanobacterial extracts on the responses of the treated plant's immune system, PR gene expression was determined in the roots of both infected plants compared with uninfected plants, 7 and 21 days after inoculation with *M. incognita* (J2s). The response was greater in plants treated with algal water extract than in plants treated with methanolic extract. The anti-nematode activity results corroborate the plant defense response findings. In particular, the water extract is capable of controlling the nematode as well as inducing the plant's immune system to resist nematode infection. Different upregulated genes were observed in both types of extract-treated plants, but the number of upregulated genes was greater in the case of water extract–treated plants. This could be because the methanolic extracts are cytotoxic to a plant's defense system. The induction activity of the algal extract on treated plants may be high in the first 3 weeks after treatment, but this effect may only last 4 weeks. Our findings confirmed that the soybean plant exhibits resistance to the nematode *M. incognita* when treated with algal extract and the plant's defense system may therefore be improved. It was reported that both PR-1 and PR-5 were highly expressed in treated plant roots as early as 1 dpt, and PR gene expression was generally comparable in treated and untreated plants at 5 dpt (Liu et al., 2006; Prasad et al., 2016). Interestingly, downregulation of the plant defense system has also been reported, specifically in the jasmonic acid pathway. The PR-1 gene in SA-treated plants is a marker of SAR induction in both roots and shoots, which may result in reduced susceptibility to RKNs. Cyanobacteria have the potential to be useful in plant protection because they can produce a variety of active compounds that can be used as herbicides, nematocides, and insecticides (Singh, 2014; Singh et al., 2016). The β-1,3-endoglucanases may play a significant role in the defense responses of plants to pathogens (Kyndt et al., 2012). Changes in PR-1 gene expression during the study period increased in response to SA treatment and this response was not different between roots and shoots. Conversely, the different responses in terms of PR-5 gene expression may indicate that different control mechanisms of this gene may exist in leaves compared to roots, as previously suggested by (Hamamouch et al., 2011). The general observation is that treatment with hot water extract of *Oscillatoria* (T1) after 7 days can protect and induce the plant immune system against invasion with *M. incognita* in the first 2 weeks after treatment, but the effect of subsequent treatments is reduced compared to the first; however, an effect was observed by the end of the third week and maybe prolonged further.

## Conclusions

It is evidenced in this study that the activity of *Oscillatoria* cyanobacterial extracts increased with increasing concentration and time of exposure. Moreover, the study proved that the isolate of cyanobacteria *Oscillatoria* sp. (isolate MT790332) extract is capable of enhancing and improving the biological and physiological properties of the treated soybean plants. Moreover, it helps in controlling the root-knot nematode *Meloidogyne incognita*, resulting in increased plant growth and resistance. Consequently, treated plants can exhibit rapid growth, high chlorophyll content, juvenile nematode mortality, and egg hatchability. Finally, we recommend that cyanobacteria *Oscillatoria* sp. (isolate MT790332) be used as a biocontrol agent against root-knot nematodes, as it can protect the plant (host) against invasion by *M. incognita* while also being economical, eco-friendly, cheap, and hazard-free.

## Data Availability Statement

The datasets presented in this study can be found in online repositories. The names of the repository/repositories and accession number(s) can be found below: https://www.ncbi.nlm.nih.gov/genbank/, MT790332.

## Author Contributions

RG and NA: data curation, investigation, and writing—original draft. RG: formal analysis, methodology, resources, validation, and visualization. MG, EE-A, RG, and DE: software. DE, MG, EE-A, and RA-S: writing—review and editing. All authors contributed to the article and approved the submitted version.

## Conflict of Interest

The authors declare that the research was conducted in the absence of any commercial or financial relationships that could be construed as a potential conflict of interest.

## Publisher's Note

All claims expressed in this article are solely those of the authors and do not necessarily represent those of their affiliated organizations, or those of the publisher, the editors and the reviewers. Any product that may be evaluated in this article, or claim that may be made by its manufacturer, is not guaranteed or endorsed by the publisher.
